# Potential Biomarkers and Drugs for Nanoparticle-Induced Cytotoxicity in the Retina: Based on Regulation of Inflammatory and Apoptotic Genes

**DOI:** 10.3390/ijerph19095664

**Published:** 2022-05-06

**Authors:** Dongli Xie, Jianchen Hu, Tong Wu, Kangli Cao, Xiaogang Luo

**Affiliations:** 1 College of Textile and Clothing Engineering, Soochow University, 199 Ren-Ai Road, Suzhou 215123, China; xdl202111@163.com (D.X.); hujianchen@suda.edu.cn (J.H.); 2 Shanghai Jing Rui Yang Industrial Co., Ltd., 3188 Xiupu Road, Pudong New Area, Shanghai 200122, China; wutong@lead-all.cn; 3 Shanghai Institute of Spacecraft Equipment, 251 Huaning Road, Shanghai 200240, China; connieckl@126.com

**Keywords:** nanoparticles, retinal injury, inflammation, apoptosis, long non-coding RNAs, competing endogenous RNAs

## Abstract

The eye is a superficial organ directly exposed to the surrounding environment. Thus, the toxicity of nanoparticle (NP) pollutants to the eye may be potentially severer relative to inner organs and needs to be monitored. However, the cytotoxic mechanisms of NPs on the eyes remain rarely reported. This study was to screen crucial genes associated with NPs-induced retinal injuries. The gene expression profiles in the retina induced by NPs [GSE49371: Au20, Au100, Si20, Si100; GSE49048: presumptive therapeutic concentration (PTC) TiO_2_, 10PTC TiO_2_] and commonly used retinal cell injury models (optic nerve injury procedure: GSE55228, GSE120257 and GSE131486; hypoxia exposure: GSE173233, GSE151610, GSE135844; H_2_O_2_ exposure: GSE122270) were obtained from the Gene Expression Omnibus database. A total of 381 differentially expressed genes (including 372 mRNAs and 9 lncRNAs) were shared between NP exposure and the optic nerve injury model when they were compared with their corresponding controls. Function enrichment analysis of these overlapped genes showed that *Tlr2*, *Crhbp*, *Ccl2*, *Cxcl10*, *Fas*, *Irf8*, *Socs3*, *Stat3*, *Gbp6*, *Casp1* and *Syk* were involved in inflammatory- and apoptotic-related processes. Protein-protein interaction network analysis revealed eight of them (*Tlr2*, *Ccl2*, *Cxcl10*, *Irf8*, *Socs3*, *Stat3*, *Casp1* and *Syk*) were hub genes. Moreover, *Socs3* could interact with upstream *Stat3* and downstream *Fas*/*Casp1*/*Ccl2*/*Cxcl10*; *Irf8* could interact with upstream *Tlr2*, *Syk* and downstream *Cxcl10*. Competing endogenous RNAs network analysis identified *Socs3*, *Irf8*, *Gdf6* and *Crhbp* could be regulated by lncRNAs and miRNAs (9330175E14Rik-mmu-miR-762-*Socs3*, 6430562O15Rik-mmu-miR-207-*Irf8*, Gm9866-mmu-miR-669b-5p-*Gdf6*, 4933406C10Rik-mmu-miR-9-5p-*Crhbp*). CMap–CTD database analyses indicated the expression levels of *Tlr2*, *Ccl2*, *Cxcl10*, *Fas*, *Irf8*, *Socs3*, *Stat3*, *Gbp6*, *Casp1* and *Syk* could be reversed by folic acid. *Crhbp* and *Gdf6* were also verified to be downregulated, while *Tlr2*, *Ccl2*, *Irf8*, *Socs3* and *Stat3* were upregulated in hypoxia/H_2_O_2_-induced retinal injury models. Hereby, our findings suggest that *Crhbp*, *Irf8*, *Socs3* and *Gdf6* as well as their upstream mRNAs, lncRNAs and miRNAs may be potential monitoring biomarkers and therapeutic targets for NP-induced retinal injuries. Folic acid supplementation may be a preventive and therapeutic approach.

## 1. Introduction

Nanoparticles (NPs), which are defined as particles with a size between 1 and 100 nm in at least one dimension, have been widely utilized in consumer products and medical applications due to their unique properties. The long-term exposure (especially for manufacturing workers or scientists) increases the possibility of NPs entering the human body through various routes (i.e., inhalation, skin absorption or ingestion) and then inducing the potential toxicity on human organs and tissues [1]. As a superficial organ, the eyes (ocular surface) are usually directly in contact with NPs dispersed in the air of the surrounding environment [2]. Thus, compared to inner organs (lung, respiratory tract, liver, kidney and brain), ocular injuries induced by toxic NPs may be severer and should be timely monitored to prevent their occurrence and progression to blindness.

Recently, there have been some toxicity studies to explore the influence of NP exposure on retinal cells as well as potential molecular mechanisms. Soderstjerna et al. used the in vitro tissue culture model of the mouse retina to observe the toxicity between 20 and 80 nm silver (Ag) and gold (Au) NPs. The results showed that compared with the control, the number of apoptotic cells in the outer nuclear layer and ganglion cell layer of mice retina was significantly increased after exposure to Ag and Au NPs (20 or 80 nm) [3]. Kalishwaralal et al. demonstrated that treatment of retinal endothelial cells with different concentrations of Ag NPs was able to block cell proliferation and migration but induce apoptosis according to the evidence of enhanced caspase-3 activity and formation of DNA fragmentation [4]. An inflammatory response with increased expressions of interleukin (IL)-6, IL-8 and tumor necrosis factor (TNF)-α was shown to be triggered after short-term exposure to graphene oxide in the eyes [5]. The study by Quan et al. revealed that Ag NPs induced apoptosis in human retinal pigment epithelium ARPE-19 cells by activating the endoplasmic reticulum stress response. Treatment with an endoplasmic reticulum stress inhibitor (4-phenylbutyric acid) significantly reduced the expression levels of caspase-3 cleavage and attenuated apoptosis in ARPE-19 cells [6]. Zinc oxide [7,8,9,10], copper NPs [11], mesoporous silica NPs [12], cerium oxide NPs [13] and PEGylated graphene oxide [14] were reported to induce apoptosis of retinal cells by elevating the levels of reactive oxygen species. The use of reactive oxygen species scavengers (reduced glutathione and N-acetylcysteine) partially restored the cell viability and alleviated retinal developmental defects [11,14]. Titanium dioxide (TiO_2_) NPs were proved to impair the inner blood-retinal barrier and retinal electrophysiology through rapid activation of ADAM17 metalloproteinase to induce ADAM17-mediated claudin-5 degradation. The use of ADAM17 chemical inhibitors (GM6001 and TAPI-2) preserved the expression levels of tight junction protein (claudin-5) and protected the integrity of the blood-retinal barrier [15]. These findings indicate that investigation of the molecular toxicological mechanisms may be beneficial for screening potential biomarkers to monitor NP-exposed individuals in order to prevent the development of retinal injury and prove potential preventive and therapeutic approaches. However, NP-related molecular toxicological mechanisms in the retina remain rarely reported.

In 2013, Jo et al. used the high-throughput microarray technique to analyze the gene expression profile of retina tissues collected from mice exposed to Au, Si and TiO_2_ NPs [16]. Although they did not detect the increased apoptotic cells in retinal tissues [16,17], we found the expression levels of several apoptosis- and inflammation-related genes were significantly changed in our preliminary analysis of this study, indicating these genes may represent potential monitoring biomarkers and therapeutic targets. Early intervention to reverse the expression levels of these genes may prevent the progression to apoptotic phenotypes. In this study, we aimed to use the raw expression profile data of Au-, Si- and TiO_2_-NPs exposure provided by Jo et al. [16] and to deeply mine crucial genes involved in the toxicity of NPs by using a series of bioinformatics analysis methods. As described above, oxidative stress damage is the common toxic effect of various NPs [7,8,9,10,11,12,13]. Thus, we hypothesize that our identified crucial genes may be altered commonly in various types of NP-induced retinal injuries and may represent underlying biomarkers for monitoring all NP-induced retinal injuries. To obtain the genes that are definitely associated with retinal injuries and apoptosis, we also collected the microarray data of commonly used retinal cell injury models (including traumatic optic nerve injury animal model, hypoxia- or H_2_O_2_-exposed animal or cell models) [18,19,20,21] and integrated with the expression profile established by Jo et al. [16].

## 2. Materials and Methods

### 2.1. Dataset Collection

The gene expression profiles in the retina exposed to NPs were obtained by searching the National Center for Biotechnology Information (NCBI) Gene Expression Omnibus (GEO) database (http://www.ncbi.nlm.nih.gov/geo/, accessed on 1 July 2021) using the keywords (“nanoparticle”) AND (“retina” or “retinal”). Following this step, only two datasets were retrieved, including GSE49371 and GSE49048 [16]. The GSE49371 dataset investigated the gene expression in the retina after phosphate buffer saline (PBS, regarded as the negative control, *n* = 12) or NPs (gold with diameters of 20 nm, Au20: *n* = 3; gold with diameters of 100 nm, Au100: *n* = 3; silicate with diameters of 20 nm, Si20: *n* = 3; silicate with diameters of 100 nm, Si100: *n* = 3) were intravitreally injected into the right vitreous cavity of 5-week-old male C57BL/6 mice for 7 days. The GSE49048 dataset [16] analyzed the gene expression in the retina after PBS (regarded as the negative control, *n* = 12) or ~25 nm TiO_2_ NPs (presumptive therapeutic concentration, PTC: 130.47 ng/mL, *n* = 3; 10 times PTC: 1.30 μg/mL, *n* = 3) were intravitreally injected into the right eye of 8-week-old male C57BL/6 mice for 7 days [16]. The PTC was determined by the cellular viability analysis in HRMECs and SNUOT-Rb1 cells and by histological analysis in mice [16]. Both GSE49371 and GSE49048 datasets were run on the platform of Agilent-026655 Whole Mouse Genome Microarray 4x44K v2 (GPL11202, Probe Name version).

Additionally, to identify whether the genes induced by NPs were associated with the retinal injury, optic nerve injury model datasets were also collected from the GEO database under accession numbers GSE55228 [22], GSE120257 [23] and GSE131486 [24]. GSE55228 [22] and GSE131486 [24] datasets explored the retinal gene expression profiles of 12-week-old mice after an optic nerve crush (ONC, *n* = 3) or sham procedure (*n* = 3) treatment by deep sequencing with Illumina Hiseq2000 platform (GPL13112). Retinal tissues were collected two days after ONC procedure in these two datasets [22,24]. The GSE120257 dataset analyzed the retinal transcriptome in 6-week-old mice that underwent (*n* = 3) or did not receive ONC (*n* = 4) treatment by high-throughput sequencing with Illumina HiSeq 3000 platform (GPL21493) [23]. Mice were sacrificed and retinas were extracted on the fourth day post-injury [23].

Moreover, hypoxia- or H_2_O_2_-exposed retinal injury model data were obtained to validate the expression levels of crucial genes in the retina, including GSE173233 (normoxia, *n* = 6; normobaric hypoxia, *n* = 24; hypobaric hypoxia, *n* = 12) [25], GSE151610 (normoxia, *n* = 3; hypoxia, *n* = 3), GSE135844 (normoxia, *n* = 3; hypoxia, *n* = 3) [26] and GSE122270 (normoxia, *n* = 3; hypoxia, *n* = 3). In the GSE173233 dataset [25], mice in normobaric hypoxia were exposed to 7% or 14% O_2_; mice in hypobaric hypoxia were exposed to the environmental air pressure of approximately 64.65 kPa, which decreased the ambient partial pressure of oxygen from 21.2 kPa (sea level) to 13.7 kPa. The GSE151610 dataset analyzed mRNA profiles of ARPE-19 cells under normoxic conditions and conditions of hypoxic stress with 0.5% O_2_. In the GSE135844 dataset [26], 7-day-old mice were placed in a 75% oxygen atmosphere or normoxic conditions. In the GSE122270 dataset, ARPE19 cells were treated with or without 10 mU/mL glucose oxidase for 3 h. The platforms of GSE173233, GSE151610, GSE135844 and GSE122270 datasets were Illumina NovaSeq 6000 (GPL24247), Illumina HiSeq 3000 (GPL21290), Affymetrix Mouse Genome 430A 2.0 Array (GPL8321) and Affymetrix Human Gene Expression Array (GPL15207), respectively.

### 2.2. Differential Expression Analysis

GSE49371 and GSE49048 datasets were two-channel microarray experiments and only the averages of normalized ratios calculated by dividing the average of normalized signal channel intensity by the average of normalized control channel intensity were provided. The differentially expressed RNAs induced by Au20, Au100, Si20, Si100 and TiO_2_ NPs were identified by calculating the *p*-value with a *t*-test and setting the average normalized ratios of three samples as the fold change (FC). The normalized expression matrix data of GSE55228, GSE120257 and GSE131486 datasets were downloaded from the GEO database. The differentially expressed RNAs between ONC and control groups in these three datasets were screened using DESeq2 (http://www.bioconductor.org/packages/release/bioc/html/DESeq2.html, accessed on 3 July 2021) package in R [27]. The differentially expressed RNAs in all datasets were selected based on the threshold of |log_2_FC| > 0.5 and *p*-value < 0.05.

The distribution of differentially expressed genes was manifested by a volcano plot for GSE49371 and GSE49048 datasets, while a heatmap was generated for GSE55228, GSE120257 and GSE131486 datasets with the ‘pheatmap’ package (v1.0.8; https://cran.r-project.org/web/packages/pheatmap, accessed on 3 July 2021). Venn diagram (http://bioinformatics.psb.ugent.be/webtools/Venn/, accessed on 5 July 2021) was applied to illustrate the overlapped genes between genes induced by NPs and ONC models. The expression differences of crucial genes in the retina were validated between hypoxia/H_2_O_2_ models and controls by a direct *t*-test with the SPSS software or online GEO2R provided by NCBI. Data management system BioMart (http://asia.ensembl.org/biomart/martview/59b47575be5aaf1f82c976009a472b38, accessed on 5 July 2021) was used to re-annotate differentially expressed RNAs to distinguish differentially expressed long non-coding RNAs (DE-lncRNAs) and protein-coding messenger RNAs (DE-mRNAs) from other RNA types.

### 2.3. Function Enrichment Analysis

Gene Ontology (GO) and Kyoto Encyclopedia of Genes and Genomes (KEGG) pathway enrichment analyses were performed to investigate the potential functions of DE-mRNAs using the Database for Annotation, Visualization, and Integrated Discovery (DAVID) online tool (v6.8; http://david.abcc.ncifcrf.gov, accessed on 8 July 2021) [28]. *p*-value < 0.05 was set as the cut-off value.

### 2.4. Construction of a Protein–Protein Interaction (PPI) Network

To screen crucial DE-mRNAs changed by NPs, a PPI network was constructed based on the protein interaction pairs (combined score ≥ 0.4) collected from the STRING (Search Tool for the Retrieval of Interacting Genes; v10.0; http://stringdb.org/, accessed on 13 July 2021) database [29]. Then, the topological characteristics of each protein in the PPI network were computed using the CytoNCA plugin in Cytoscape software (http://apps.cytoscape.org/apps/cytonca, accessed on 13 July 2021) [30,31], including the degree centrality (DC, measure of the number of interactive neighbors of a protein), eigenvector centrality (EC, measure of the component of the principal eigenvector of adjacency matrix), local average connectivity (LAC, measure of the local connectivity of its neighbors), betweenness centrality (BC, measure of the number of shortest paths going through a protein) and closeness centrality (CC, measure of the average distance from a protein to all other proteins). The proteins that had higher levels of these topological parameters were considered to be important for diseases. The proteins ranked in the top 60 according to the values calculated for each centrality measure were selected as hub genes.

Furthermore, function-related modules were also extracted from the PPI network using the Molecular Complex Detection (MCODE; v1.4.2, http://apps.cytoscape.org/apps/mcode, accessed on 13 July 2021) plugin of Cytoscape software with the following setting parameters: MCODE score > 4, degree cut-off = 2, node score cut-off = 0.2, k-core = 2 and max depth = 100 [32]. The hub genes included in modules were further confirmed to be crucial.

### 2.5. Construction of a Competing Endogenous RNAs (ceRNAs) Regulatory Network

LncRNAs were reported to function as ceRNAs to competitively bind with microRNAs (miRNAs) and then influence the negative regulatory roles of miRNAs on the expressions of targeted mRNAs [33]. To screen crucial DE-lncRNAs and reveal their functions, a ceRNA network was constructed based on the interaction pairs of lncRNAs-miRNAs and miRNAs-mRNAs. DIANA-LncBase (v2.0; http://carolina.imis.athena-innovation.gr/diana_tools/web/index.php?r=lncbasev2/index-predicted, accessed on 16 July 2021) [34] and starbase (v3.0; http://starbase.sysu.edu.cn, accessed on 16 July 2021) databases were used to predict the interacted miRNAs for DE-lncRNAs. miRwalk database (v2.0; http://www.zmf.umm.uni-heidelberg.de/apps/zmf/mirwalk2, accessed on 16 July 2021) that contained 12 prediction programs [35] was used to predict the target mRNAs regulated by DE-lncRNA-interacted miRNAs. Only the miRNA-target gene interaction pairs that were predicted by more than 8 prediction programs were selected. Additionally, target mRNAs were subsequently overlapped with DE-mRNAs. The expression direction of DE-lncRNAs and DE-mRNAs that interacted with miRNAs should be consistent (that is, both upregulated or downregulated). The ceRNA network was constructed and visualized using Cytoscape (v3.6.1; www.cytoscape.org/, accessed on 16 July 2021).

### 2.6. Small Molecule Drug Analysis

To predict potential drugs for prevention and treatment of NP-induced retinal injury, Connectivity Map (CMap, https://portals.broadinstitute.org/cmap/, accessed on 18 July 2021) and Comparative Toxicogenomics Database (CTD, http://ctdbase.org, accessed on 18 July 2021) analyses were performed. The upregulated and downregulated DE-mRNAs were uploaded as the gene signature to query the CMap database, after which a series of small molecule drugs with enrichment scores ranging from −1 to 1 could be obtained. The small molecule drugs with negative connectivity scores and *p*-value < 0.05 were considered to reverse the expression direction of the query gene signature and may be therapeutic drugs. The small molecule drugs identified by CMap were then used as the keywords to search the CTD to collect the chemical-gene interaction pairs. The drugs that specifically targeted the DE-mRNAs were believed to be particularly important. The structures of the candidate drugs were obtained from the DrugBank database (https://www.drugbank.ca/, accessed on 18 July 2021). The gene-drug interaction networks were visualized using Cytoscape software.

## 3. Results

### 3.1. Differential Expression Analysis

**Figure 1:**
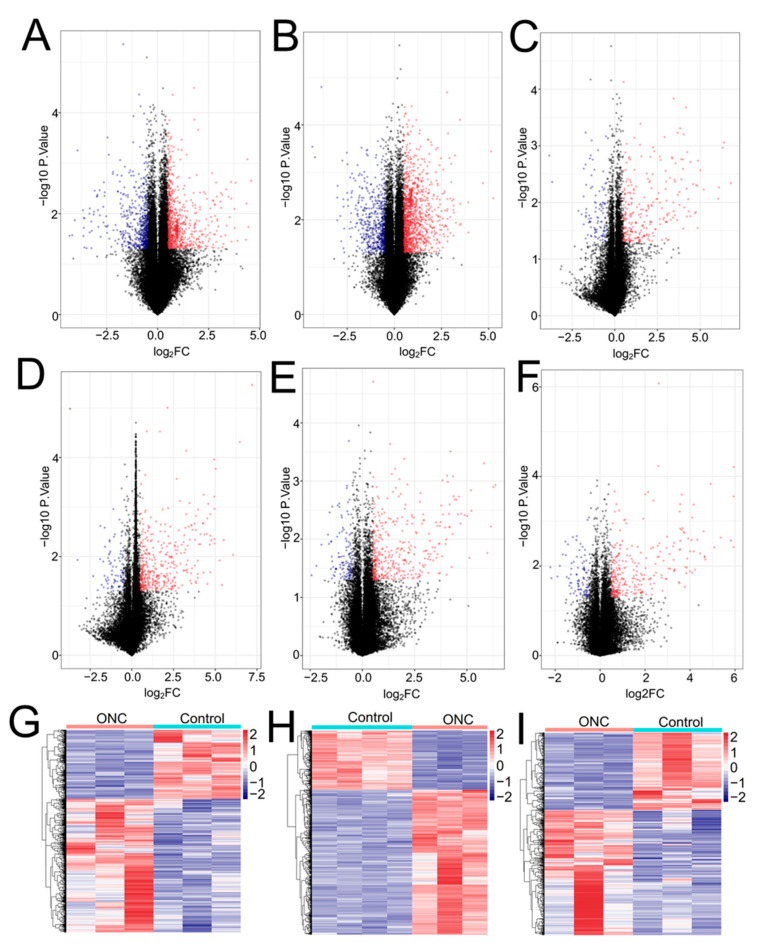
Identification of differentially expressed RNAs. (**A**) a volcano plot to show the differentially expressed RNAs of the GSE49048 dataset with PTC TiO_2_ NP exposure; (**B**) a volcano plot to show the differentially expressed RNAs of the GSE49048 dataset with 10PTC TiO_2_ NP exposure; (**C**) a volcano plot to show the differentially expressed RNAs of the GSE49371 dataset with Au20 NP exposure; (**D**) a volcano plot to show the differentially expressed RNAs of the GSE49371 dataset with Au100 NP exposure; (**E**) a volcano plot to show the differentially expressed RNAs of the GSE49371 dataset with Si20 NP exposure; (**F**) a volcano plot to show the differentially expressed RNAs of the GSE49371 dataset with Au100 NP exposure; (**G**) a heat map to show the differentially expressed RNAs of the GSE55228 dataset undergoing ONC procedures; (**H**), a heat map to show the differentially expressed RNAs of the GSE120257 dataset undergoing ONC procedures; (**I**) a heat map to show the differentially expressed RNAs of the GSE131486 dataset undergoing ONC procedures. Blue dots in the volcano plots are downregulated genes; red dots in the volcano plots are upregulated genes; blue in the heatmap indicates high expressed genes; red in the heatmap indicates low expressed genes; FC, fold change; ONC, optic nerve crush; PTC: presumptive therapeutic concentration; NPs: nanoparticles; Ag: silver; Au: gold; Si: silicate.

In the GSE49048 dataset, a total of 1346 (794 upregulated; 552 downregulated) (Figure 1A) and 1852 (1266 upregulated; 586 downregulated) (Figure 1B) RNAs were considered to be differentially expressed in the retina of mice undergoing PTC and 10PTC TiO_2_ NP exposure, respectively. In the GSE49371 dataset, a total of 365 (263 upregulated; 102 downregulated) (Figure 1C), 503 (416 upregulated; 87 downregulated) (Figure 1D), 514 (391 upregulated; 123 downregulated) (Figure 1E) and 392 (297 upregulated; 95 downregulated) (Figure 1F) differentially expressed RNAs were, respectively, found between Au20-, Au100-, Si20-, Si100- and PBS-exposed retina.

Compared with the control group, the GSE55228 dataset analysis identified 303 upregulated and 587 downregulated RNAs in the retina of ONC model mice (Figure 1G); a total of 520 upregulated and 212 downregulated RNAs were screened in the GSE120257 dataset between the ONC and control groups (Figure 1H); for the GSE131486 dataset, 322 RNAs were shown to be upregulated and 199 were downregulated in the ONC model retina in comparison with controls (Figure 1I).

**Figure 2:**
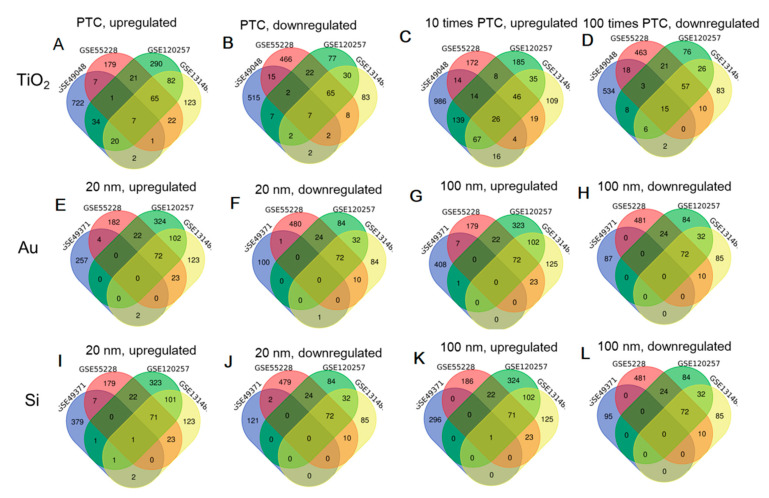
Venn diagrams to show the overlapped genes between NP exposure and ONC treatment. (**A**) overlapped upregulated genes between PTC TiO_2_ NPs of GSE49048 and GSE55228-GSE120257-GSE131486; (**B**) downregulated upregulated genes between PTC TiO_2_ NPs of GSE49048 and GSE55228-GSE120257-GSE131486; (**C**) overlapped upregulated genes between 10PTC TiO_2_ NPs of GSE49048 and GSE55228-GSE120257-GSE131486; (**D**) overlapped downregulated genes between 10PTC TiO_2_ NPs of GSE49048 and GSE55228-GSE120257-GSE131486; (**E**) overlapped upregulated genes between Au20 NPs of GSE49371 and GSE55228-GSE120257-GSE131486; (**F**) overlapped downregulated genes between Au20 NPs of GSE49371 and GSE55228-GSE120257-GSE131486; (**G**) overlapped upregulated genes between Au100 NPs of GSE49371 and GSE55228-GSE120257-GSE131486; (**H**) overlapped downregulated genes between Au100 NPs of GSE49371 and GSE55228-GSE120257-GSE131486; (**I**) overlapped upregulated genes between Si20 NPs of GSE49371 and GSE55228-GSE120257-GSE131486; (**J**) overlapped downregulated genes between Si20 NPs of GSE49371 and GSE55228-GSE120257-GSE131486; (**K**) overlapped upregulated genes between Si100 NPs of GSE49371 and GSE55228-GSE120257-GSE131486; (**L**) overlapped downregulated genes between Si100 NPs of GSE49371 and GSE55228-GSE120257-GSE131486. PTC: presumptive therapeutic concentration; NPs: nanoparticles; Ag: silver; Au: gold; Si: silicate.

The ONC procedure is the most commonly used retinal damage model [21]. To obtain crucial genes involved in NP-induced retinal injury, we selected the shared differentially expressed RNAs between GSE49048-GSE49371 and GSE55228-GSE120257-GSE131486 datasets. As a result, Venn diagram showed that there were 16, 62 and 30 commonly upregulated genes (Figure 2A); 26, 18 and 13 commonly downregulated genes (Figure 2B) between PTC TiO_2_ NP exposure in the GSE49048 dataset with ONC models of GSE55228, GSE120257, GSE131486 datasets, respectively; a total of 58, 246 and 113 commonly upregulated genes (Figure 2C); 36, 32 and 23 commonly downregulated genes (Figure 2D) were found between 10PTC TiO_2_ NP exposure in the GSE49048 dataset with ONC models of GSE55228, GSE120257, GSE131486 datasets, respectively; there were four and two commonly upregulated genes (Figure 2E); each one commonly downregulated gene (Figure 2F) between Au20 exposure in the GSE49371 dataset with ONC models of GSE55228 and GSE131486 datasets, respectively; a total of seven and one commonly upregulated genes (Figure 2G) were found between Au100 exposure in the GSE49371 dataset with ONC models of GSE55228 and GSE120257 datasets, respectively; there were eight, three and four commonly upregulated genes (Figure 2I) between Si20 exposure in the GSE49371 dataset with ONC models of GSE55228, GSE120257, GSE131486 datasets, respectively; only two commonly downregulated genes (Figure 2J) were identified between Si20 exposure in the GSE49371 dataset with ONC models of the GSE55228 dataset; each one upregulated gene (Figure 2K) was shown to be shared between Si100 exposure in the GSE49371 dataset with ONC models of GSE55228, GSE120257, GSE131486 datasets, respectively. No overlapped downregulated genes were identified when Au100 (Figure 2H) and Si100 (Figure 2L) exposure were compared with ONC models. Ultimately, 389 shared RNAs were obtained, including 372 mRNAs, 9 lncRNAs (6430562O15Rik, 9330175E14Rik, Gm833, Gm10030, 0610009B14Rik, Gm9866, Dbpht2, 4933406C10Rik, D030047H15Rik) and 8 other RNA types. These 372 DE-mRNAs and 9 DE-lncRNAs were used for the following analysis (Table 1).

### 3.2. Function Enrichment Analysis

**Figure 3:**
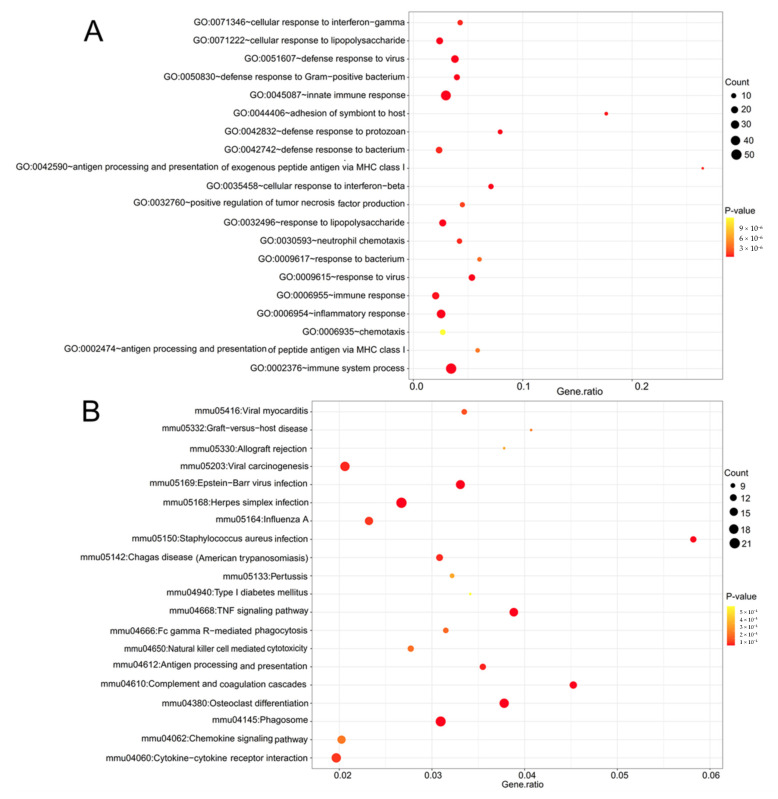
Function enrichment for the overlapped differentially expressed mRNAs between GSE49048-GSE49371 and GSE55228-GSE120257-GSE131486 datasets. (**A**) GO term; (**B**) KEGG pathways. Only top 20 are shown. GO: Gene Ontology; KEGG: Kyoto Encyclopedia of Genes and Genomes.

These 372 DE-mRNAs were uploaded into the DAVID database to predict their functions. The results showed that 170 significant GO biological process terms and 40 significant KEGG pathways were enriched. As shown in Figure 3, these genes were mainly involved in inflammation-related processes, such as GO:0045087~innate immune response (IFIH1, TLR2, SYK), GO:0006954~inflammatory response (CRHBP, CCL2, CXCL10, FAS, TLR2), GO:0006955~immune response (CXCL10, CCL2, FAS, IRF8, TLR2), GO:0071346~cellular response to interferon-gamma (CCL2), GO:0006935~chemotaxis (CXCL10, CCL2), mmu04668:TNF signaling pathway (CXCL10, SOCS3, CCL2, FAS), mmu04060:Cytokine-cytokine receptor interaction (CX3CR1, CXCL9, CXCL10, CCL2, FAS) and mmu04062:Chemokine signaling pathway (STAT3, CXCL10, CCL2). Furthermore, apoptosis-related processes were also included, such as GO:0043066~negative regulation of apoptotic process (STAT3, SOCS3), GO:0043065~positive regulation of apoptotic process (CASP1, FAS), GO:0006915~apoptotic process (CASP1, FAS, GDF6), GO:0042981~regulation of apoptotic process (CASP1, FAS, GDF6), GO:0043524~negative regulation of neuron apoptotic process (CCL2) (Table 2) and mmu04210:Apoptosis (FAS) (Table 3). Importantly, some genes enriched in inflammatory and apoptotic processes were shared, suggesting NP-induced retinal injuries may be associated with inflammation-mediated apoptosis of retinal neurons.

### 3.3. PPI Network

Totally, 300 of the 372 DE-mRNAs were shown to interact with each other to form 2603 PPI pairs (e.g., *Socs3*-*Stat3*/*Casp1*/*Fas*/*Ccl2*/*Cxcl10*, *Irf8*-*Tlr2*/*Syk*/*Cxcl10*) that were used to construct the PPI network. After calculation of 5 topological features for each protein in the PPI network, 116 genes were ranked in the top 60 genes (20%), indicating they were potential hub genes (Table 4). Among them, inflammation- or apoptosis-related *Tlr2*, *Irf8*, *Ifih1* and *Cxcl10* were listed in the top 60 genes of all topological features (DC, BC, CC, DC and EC); *Stat3*, *Ccl2* and *Casp1* were listed in the top 60 genes based on BC, CC, DC and EC ranking; Syk was considered as a hub gene according to CC, DC and EC ranking; *Socs3* was believed as a hub gene according to BC and CC ranking.

**Figure 4:**
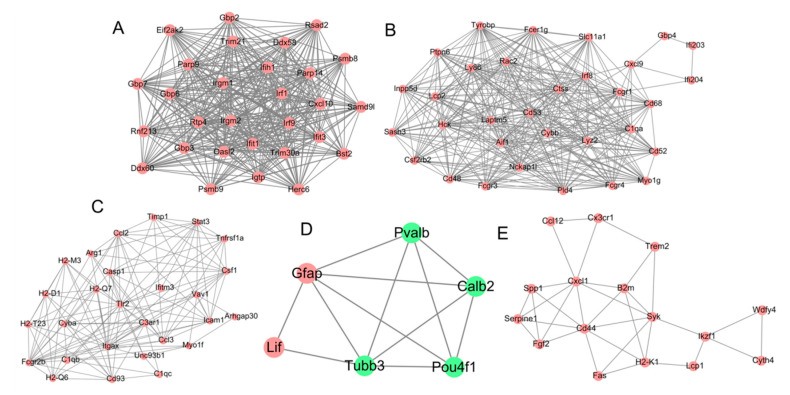
Modules extracted from the protein–protein interaction network. (**A**) module 1; (**B**) module 2; (**C**) module 3; (**D**) module 4; (**E**) module 5. Red, upregulated genes; green, downregulated genes.

Furthermore, five significant modules were extracted from the PPI network (Table 5; Figure 4). The above inflammation- or apoptosis-related hub genes were also contained in these five modules (*Ifih1*, *Cxcl10* in module 1; *Irf8* in module 2; *Stat3*, *Tlr2*, *Casp1*, *Ccl2* in module 3; *Syk* in module 5), further explaining their importance for NP-induced retinal injuries.

### 3.4. Construction of a ceRNA Network

**Figure 5:**
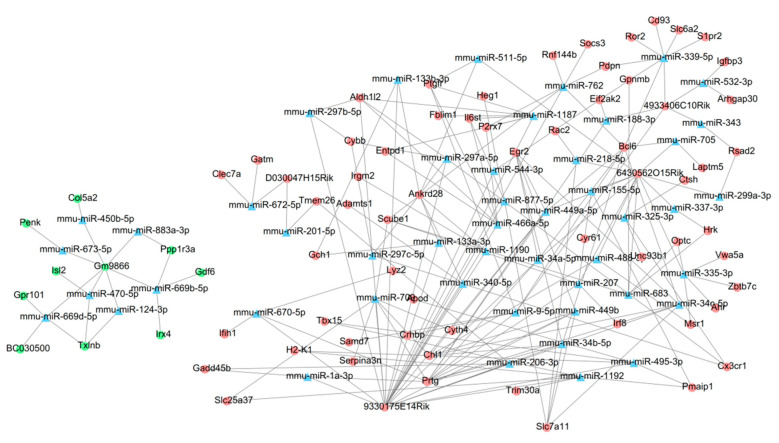
A ceRNA network among differentially expressed mRNAs, lncRNAs and miRNAs. Circle indicates the mRNAs; hexagon indicates the lncRNAs; triangle indicates the miRNAs; Red, upregulated genes; green, downregulated genes; blue, not differentially expressed. ceRNA, competing endogenous RNAs.

The DIANA-LncBase and starbase databases predicted that 7 lncRNAs (0610009B14Rik, 4933406C10Rik, D030047H15Rik, 6430562O15Rik, Gm9866, 9330175E14Rik, Gm833) could interact with 278 miRNAs. The miRwalk2.0 database predicted 62 of these 278 miRNAs could interact with 105 target genes according to more than 8 prediction programs. After screening the DE-lncRNAs and DE-mRNAs with the consistent expression directions, regulatory relationship pairs among 5 lncRNAs, 49 miRNAs and 69 mRNAs were ultimately obtained, which was used to construct the ceRNA network (Figure 5). Among them, *Socs3*, *Irf8* and *Ifih1* were overlapped with hub genes identified in the PPI network, forming the following interaction axes: 9330175E14Rik-mmu-miR-762-*Socs3*, 6430562O15Rik-mmu-miR-207-*Irf8* and 9330175E14Rik-mmu-miR-670-5p-*Ifih1*. These findings suggested that these ceRNA axes were important for NP-induced retinal injuries. The Gm9866-mmu-miR-669b-5p-*Gdf6* and 4933406C10Rik-mmu-miR-9-5p-*Crhbp* ceRNA axes were also crucial because *Gdf6* and *Crhbp* were enriched in apoptotic or inflammatory processes, respectively. Moreover, *Gdf6* and *Crhbp* have limited overlapped genes between Au or Si NP exposure and ONC models.

### 3.5. Identification of Small Molecule Drugs

**Figure 6:**
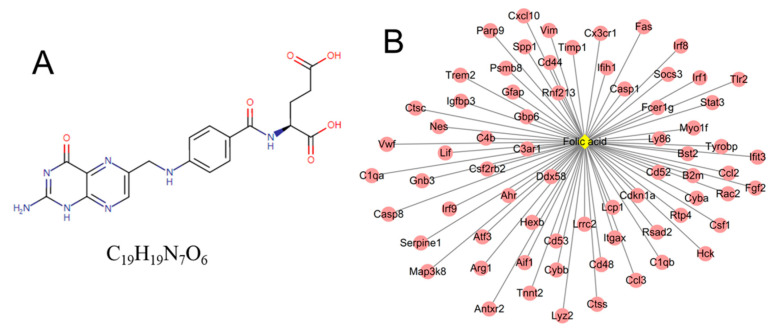
The target relationships between folic acid and genes. (**A**) the structure of folic acid; (**B**) the regulatory network between folic acid and genes. Red, upregulated genes.

A total of 20 small molecules were found to have a negative enrichment score and a *p*-value < 0.05 (Table 6), indicating they may be candidate drugs for the prevention and treatment of NP-induced retinal injuries. Among them, folic acid could target 69 hub DE-mRNAs (including *Casp1*, *Ccl2*, *Cxcl10*, *Fas*, *Gbp6*, *Ifih1*, *Irf8*, *Socs3*, *Stat3*, *Tlr2*, *Syk*) (Figure 6). Thus, folic acid tablets or folic acid-enriched foods should be properly supplemented for NP-exposed individuals to prevent the development of retinal injuries.

### 3.6. Validation of Crucial Genes in Hypoxia/H_2_O_2_-Induced Retinal Injury Models

**Figure 7:**
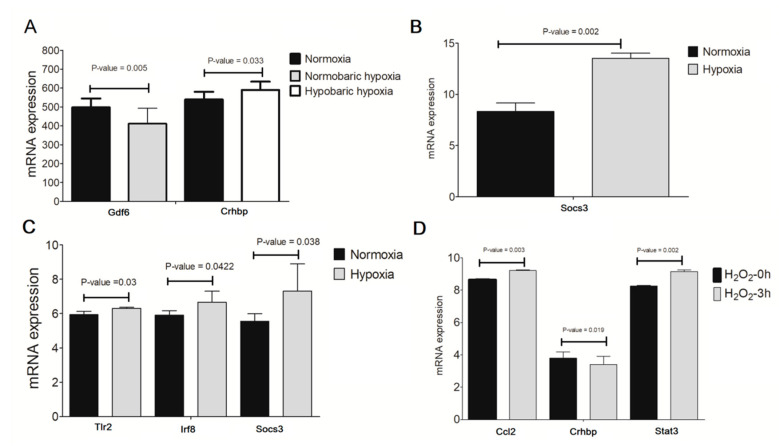
Validation of the expression levels of crucial genes in hypoxia/H_2_O_2_-induced retinal injury models. (**A**) GSE173233 dataset; (**B**) GSE151610 dataset; (**C**) GSE135844 dataset; (**D**) GSE122270 dataset. h, hour.

The expression levels of crucial genes (including *Casp1*, *Ccl2*, *Cxcl10*, *Fas*, *Gbp6*, *Ifih1*, *Irf8*, *Socs3*, *Stat3*, *Tlr2*, *Syk* and *Crhbp*) obtained after the above-integrated analyses were also validated in hypoxia/H_2_O_2_-induced retinal injury models. In line with NP exposure and ONC, *Gbp6* and *Crhbp* were further confirmed to be lowly expressed (Figure 7A,D), while *Socs3*, *Tlr2*, *Irf8*, *Ccl2* and *Stat3* were highly expressed in hypoxia/H_2_O_2_-induced retinal injury models compared with controls (Figure 7B–D).

## 4. Discussion

Although there are anatomical barriers on the eyeballs, only the airborne large-sized particles could be blocked from the ocular surface by blinking and tear film, while small-sized particulate matter NPs penetrate the barriers of the ocular surface and reach the posterior segments of the eyes [2,36,37]. Once entering the eyes, NPs may subsequently induce cellular toxicity in the lens, retina, optic nerve, and macula by stimulating inflammatory cell infiltration and cell apoptosis [2,36,37], ultimately leading to the development of ocular diseases. Furthermore, NPs are commonly utilized as drug delivery agents, which gives them the chance to be directly injected into the retina or reach the retina by impairing and crossing the blood-retinal barrier after systemic administration [38]. Hereby, the toxic mechanisms of NPs for retinal injuries should be given high attention. In the present study, we integrated the expression profile data of NP exposure and ONC models and identified that NP exposure triggered expression changes in 12 inflammation- or apoptosis-related genes (*Ifih1*, *Tlr2*, *Crhbp*, *Ccl2*, *Cxcl10*, *Fas*, *Irf8*, *Socs3*, *Stat3*, *Gbp6*, *Casp1*, *Syk*) in the retina. Among them, 9 (*Ifih1*, *Cxcl10*, *Irf8*, *Stat3*, *Tlr2*, *Casp1*, *Ccl2*, *Syk*, *Socs3*) were revealed to be hub genes according to PPI network and module analyses; 5 (*Socs3*, *Irf8*, *Ifih1*, *Gdf6*, *Crhbp*) were contained in the ceRNA network; 11 (*Casp1*, *Ccl2*, *Cxcl10*, *Fas*, *Gbp6*, *Ifih1*, *Irf8*, *Socs3*, *Stat3*, *Tlr2*, *Syk*) could be reversed by folic acid; 7 (*Gbp6*, *Crhbp*, *Socs3*, *Tlr2*, *Irf8*, *Ccl2*, *Stat3*) were validated to be differentially expressed in hypoxia/H_2_O_2_-induced retinal injury models. Two (*Socs3* and *Irf8*) were the overlapped genes of all these procedures. Thus, we considered Socs3 and Irf8 to be particularly important monitoring biomarkers and therapeutic targets associated with NP-induced retinal injuries.

There have been several studies to show that genetic deletion of suppressor of cytokine signaling 3 (SOCS3) enhances robust and sustained axon regeneration in the optic nerve of mice after ONC, while overexpression of SOCS3 results in almost complete regeneration failure of retinal ganglion cells [39,40,41,42]. In vitro studies demonstrated that the administration of SOCS3 markedly promoted the apoptosis of retinal pigment epithelial cells by increasing the expression levels of inflammatory mediators (IL-6 and TNF-α) [43]. Knockdown of SOCS3 significantly increased anti-apoptotic proteins (Akt, Bcl-xL), while decreased pro-apoptotic proteins (cytochrome C, Bax, caspase 3) in retinal endothelial cells [44]. The SOCS3 upregulated in degenerative retinal tissues resulted from the long-term activation of the signal transducer and activator of the transcription 3 (STAT3) pathway [45]. Furthermore, long-term exposure to TiO_2_ NPs was reported to lead to the infiltration of inflammatory cells and hepatocyte apoptosis or necrosis by upregulating the expression levels of STAT3 [46]. Thus, the hypothesis that NP exposure promoted the apoptosis of retinal cells by activating the STAT3-SOCS3-inflammation pathway may be believable. In line with these studies, we also observed that SOCS3 could interact with STAT3 as well as pro-apoptotic genes FAS (Fas cell surface death receptor) [47], CASP1 (caspase 1) [48], pro-inflammatory CCL2 (C-C motif chemokine ligand 2, also known as Mcp-1) [49] and CXCL10 (C-X-C motif chemokine ligand 10) [50] in the retina, confirmed by previous studies. Similar to the expected results, all of these genes were found to be upregulated in retinal tissues after TiO_2_ NP exposure and the ONC procedure in our study. SOCS3, STAT3 and CCL2 were also validated to be highly expressed in hypoxia/H_2_O_2_-induced retinal injury models.

A previous study observed that loss of interferon regulatory factor 8 (IRF8) in retinal microglial cells and neurons protected the mice from the development of uveitis (with fewer numbers of inflammatory cells in the vitreous and less retinal infolding) [51]. Retinal degeneration (showing increased retinal thickness) was detected to be alleviated when the expression levels of IRF8 were inhibited by immunomodulatory agents [52,53]. Mechanistic investigation showed that the amelioration roles of retina-specific Irf8-deficiency for retinal diseases were ascribed to enhance the production of anti-inflammatory cytokines (IL-10, IL-27 and IL-35) and reduce the expression levels of pro-inflammatory IL-17 in the retina [51], while IRF8 was suggested as a downstream target gene of spleen tyrosine kinase (SYK) [54] and toll-like receptor (TLR)-4 was an adaptor protein of SYK [55] in the pathogenesis of ocular injuries. Cui et al. [56] and Hong et al. [57] proved that TiO_2_ NP exposure induced inflammatory histopathological changes and apoptosis of hepatocytes or spermatogenic/Sertoli cells by significantly increasing the mRNA and protein expression levels of TLR2, TLR3 and TLR4, respectively. Ag NP-mediated apoptosis in chondrocytes and periodontal ligaments was implied to be reduced after treatment with TLR2 siRNAs and antibodies [58]. Zinc oxide NPs were indicated to have significant adjuvant effects to induce inflammatory responses via activation of SYK and TLR signaling pathways [59]. In agreement with these studies, we found that IRF8 could interact with SYK, TLR2 and CXC10. All of these genes were upregulated in retinal tissues after TiO_2_ NP exposure and the ONC procedure in our study. IRF8 and TLR2 were also validated to be highly expressed in hypoxia/H_2_O_2_-induced retinal injury models. Therefore, NP exposure may stimulate visual impairments by activation of the following pathway: TLRs→SYK→IRF8→CXCL10→inflammation→apoptosis→retinal injury.

Existing evidence supports that depletion of growth differentiation factor 6 (GDF6, also known as BMP13) in embryos results in a reduction in the eye size, a loss of mature neurons and an increase in cell death [60]. Overexpression of CRHBP (corticotropin-releasing hormone-binding protein) was shown to promote apoptosis in renal cell carcinoma via activating the nuclear factor (NF)-κB signaling pathway [61]. Similar to these studies, we also identified that GDF6 was downregulated while CRHBP was upregulated to participate in inflammation-mediated apoptosis in retinal tissues after TiO_2_, Au, Si NP exposure, hypoxia/H_2_O_2_ and ONC procedures.

Although there had been studies to investigate the molecular mechanisms for NP exposure by the high-throughput technology, all of them focused on the protein-coding mRNAs [10,14] and lncRNAs remained underexplored. In the present study, we identified 9 crucial lncRNAs (6430562O15Rik, 9330175E14Rik, Gm833, Gm10030, 0610009B14Rik, Gm9866, Dbpht2, 4933406C10Rik, D030047H15Rik) associated with NP exposure. Most of them were not reported previously, except for Gm9866, which was demonstrated to be highly expressed in cardiac hypertrophy model mice [62]. This study indicated that Gm9866 may be downregulated if cell apoptosis predominated, which seemed to be consistent with our results of TiO_2_ NP exposure. To imply the possible functions of these lncRNAs, we constructed a ceRNA network. Consequently, 9330175E14Rik-mmu-miR-762-*Socs3*, 6430562O15Rik-mmu-miR-207-*Irf8*, Gm9866-mmu-miR-669b-5p-*Gdf6*, and 4933406C10Rik-mmu-miR-9-5p-*Crhbp* ceRNA axes were obtained. Thus, upregulated 9330175E14Rik, 6430562O15Rik, 4933406C10Rik and downregulated Gm9866 may exert pro-inflammatory and pro-apoptotic roles by leading to the upregulation of *Socs3*, *Irf8*, *Crhbp*, and downregulation of *Gdf6*, respectively. Although miRNAs in ceRNAs were not differentially expressed by NP exposure, their roles in other diseases may indirectly indicate their functions. For example, Gao et al. found that treatment with miR-762 inhibitors significantly inhibited the proliferation of retinal progenitor cells [63]. Tao et al. reported that miR-207 mimics protected against autophagic cell death after ischemic stroke and attenuated neurological deficit scores and infarct volumes [64]. Chi et al. observed that enforced expression of miR-9 increased cell viability, inhibited cell apoptosis and inactivated endoplasmic reticulum stress in oxygen-glucose deprivation neurons [65]. Overexpression of miR-669b promoted the secretion of pro-inflammatory TNF-α in CD4+ T cells [66]. Accordingly, we speculated that miR-762, miR-207 and miR-9 were downregulated, while miR-669b was upregulated in retinal cells with NP exposure to promote inflammation and cell apoptosis, which conformed to our ceRNA theory according to the expression trend of miRNA-interacted lncRNAs and mRNAs.

Additionally, we identified that folic acid may be an underlying drug to treat NP-induced retinal injuries by reversing the above inflammatory and apoptotic genes. Our results were consistent with previous studies that demonstrated the protective roles of folic acid in retinal injuries induced by other factors and its influence on the expression levels of some genes in other inflammatory diseases. For example, Muralidharan et al. found that 2% ethanol exposure was shown to increase retinal cell death in zebrafish, while supplementation of 75 µM folic acid rescued retinal photoreceptor and ganglion cell differentiation defects [67]. Iskandar et al. observed that intraperitoneal treatment of adult rats with folic acid (80 µg/kg) for two weeks significantly improved the regrowth of retinal ganglion cells and enhanced neurological recovery from a spinal cord contusion injury [68]. Ma et al. verified that folic acid supplementation (40 μg/mL) repressed a hypoxia-induced inflammatory response via downregulating the activity of STAT3 and decreasing the expression levels of NF-κB p65 protein in human promyelomonocytic cells [69]. The study by Cui et al. showed that folic acid (2.1 mg/kg diet) reduced the atherosclerotic lesion size of mice by inhibiting the expression levels of CCL2 [70].

Some limitations should be acknowledged. First, this study only used the public transcriptome data of retinal tissues after Au, Si, and TiO_2_ exposure in mice to identify the molecular toxicological mechanisms of NPs. To confirm whether they represent underlying biomarkers for monitoring all NP-induced retinal injuries, the expression levels of our identified genes still need to be detected in animal or cell models by exposing them to different sizes, concentrations, chemical modifications and other characteristics of various NPs and collecting different tissue (e.g., cornea, conjunctiva, lens, sclera, choroid and retina) and cell types (e.g., epithelial, endothelial, ganglion cells) of eyes [2,5]. Second, our identified crucial genes were predicted to be involved in NP-induced retinal injury by regulation of inflammation and apoptosis. Thus, overexpression or silencing wet experiments should be scheduled in vitro and in vivo and then validated for their influence on cell proliferation (CCK8 assay), apoptosis (annexin V/PI staining, TUNEL), the release of inflammatory cytokines (enzyme-linked immunosorbent assay) and visual functions (symptom observation, fluorescein staining, tear secretion, electroretinogram and iris angiography) [5,12,71,72]. Third, the PPI and ceRNA regulatory mechanisms among genes need to be verified by immunoprecipitation or a dual-luciferase reporter assay. Fourth, folic acid was suggested to be a potential drug for preventing and treating NP-induced eye injury. Which dose may be most beneficial needs to be first determined by in vitro and in vivo experiments and then converted to a dose for humans and recommended for occupational exposure to NPs.

## 5. Conclusions

In summary, our findings suggest that *Socs3*, *Irf8*, *Crhbp* and *Gdf6* as well as the upstream mRNAs (*Stat3*, *Tlr2*-*Syk*), lncRNAs (9330175E14Rik, 6430562O15Rik, 4933406C10Rik and Gm9866) and miRNAs (miR-762, miR-207, miR-9 and miR-669b) that regulate these genes may be potential monitoring biomarkers and therapeutic targets associated with retinal injuries induced by NPs. They may participate in NP-induced visual impairments by activating inflammatory and apoptotic pathways. Folic acid nutrient supplementation may be a preventive and therapeutic approach for NP-induced retinal injuries.

## Figures and Tables

**Table 1:** Crucial genes changed by nanoparticles to induce the retinal injury.

Treatment	Gene Symbol	GSE49048		GSE55228		GSE120257		GSE131486	
		Log_2_FC	*p*-Value	Log_2_FC	*p*-Value	Log2FC	*p*-Value	Log_2_FC	*p*-Value
TiO_2_ vs. control (PTC)	Gm9866	−0.50	3.94 × 10^2^					−0.57	2.74 × 10^2^
	*Cx3cr1*	1.44	3.97 × 10^2^			0.85	6.09 × 10^13^	0.62	1.47 × 10^2^
	*Stat3*	1.00	2.98 × 10^2^			1.04	4.73 × 10^51^	0.63	2.83 × 10^2^
	*Aif1*	1.35	4.44 × 10^2^			0.78	1.90 × 10^6^		
	*Cxcl9*	0.65	3.49 × 10^2^			0.91	4.91 × 10^11^		
TiO_2_ vs. control (100 PTC)	6430562O15Rik	0.54	1.32 × 10^3^			0.77	1.04 × 10^7^		
	9330175E14Rik	1.24	3.92 × 10^2^			0.62	3.30 × 10^6^		
	*Tlr2*	1.08	1.60 × 10^3^	2.38	1.55 × 10^3^	1.14	3.06 × 101^2^	1.54	4.20 × 10^2^
	*C1qa*	1.44	1.59 × 10^3^	0.64	5.26 × 10^3^	1.73	5.75 × 10^51^		
	*Cx3cr1*	1.16	4.06 × 10^3^			0.85	6.09 × 10^13^	0.62	1.47 × 10^2^
	*Irf1*	0.66	2.83 × 10^2^			1.25	5.84 × 10^30^	0.57	1.01 × 10^2^
	*Irf8*	1.39	6.97 × 10^4^			1.05	3.01 × 10^13^	1.07	4.07 × 10^3^
	*Cxcl10*	2.43	8.73 × 10^3^			1.39	4.11 × 10^18^	1.90	1.31 × 10^2^
	*Socs3*	1.36	1.3 × 10^4^			1.61	3.81 × 10^38^	1.08	2.43 × 10^2^
	*Ccl2*	1.60	3.24 × 10^3^					5.63	7.48 × 10^3^
	*Stat3*	1.03	9.11 × 10^4^			1.04	4.73 × 10^51^	0.63	2.83 × 10^2^
	*Ifih1*	0.91	1.10× 10^2^					0.73	7.64 × 10^4^
	*Aif1*	1.17	8.88 × 10^3^			0.78	1.90 × 10^6^		
	*Fas*	1.00	9.77 × 10^3^			0.70	1.63 × 10^5^		
	*Casp1*	1.19	3.2 × 10^4^			0.56	7.35 × 10^5^		
	*Cxcl9*	0.61	1.37 × 10^3^			0.91	4.91 × 10^11^		
	*Pou4f1*	−0.62	1.00× 10^2^	−0.70	2.27 × 10^7^	−1.50	3.19 × 10^55^	−1.48	1.78 × 10^10^
Au20 vs. control	4933406C10Rik	0.73	3.32× 10^2^	0.96	9.35 × 10^3^				
	*Crhbp*	1.22	4.29× 10^2^	0.61	2.44 × 10^2^				
	*Gdf6*	−0.98	1.20 × 10^3^	−1.14	4.97 × 10^2^				
Au100 vs. control	4933406C10Rik	0.74	385× 10^2^	0.96	9.35 × 10^3^				
	*Crhbp*	2.10	3.40× 10^2^	0.61	2.44 × 10^2^				
	D030047H15Rik	0.56	5.06 × 10^3^	0.87	2.08 × 10^2^				
Si20 vs. control	4933406C10Rik	0.61	1.60× 10^2^	0.96	9.35 × 10^3^				
	*Crhbp*	1.74	2.73× 10^2^	0.61	2.44 × 10^2^				

FC: fold change; PTC: presumptive therapeutic concentration; TiO_2_: titanium dioxide; Au20: gold with diameters of 20 nm; Au100: gold with diameters of 100 nm; Si20: silicate with diameters of 20 nm; Si100: silicate with diameters of 100 nm.

**Table 2:** GO term enrichment results.

Term	*p*-Value	Genes
GO:0002376~immune system process	3.04 × 10^27^	IFITM3, H2-T23, SPON2, CD84, CSF1, H2-K1, AHR, IFI30, IFIT1, IFIT3, IFIH1, MAP3K8, LGALS9, JAK3, B2M, OASL2, HERC6, RSAD2, SYK, DDX58, TAP1, FCGR1, NAIP2, HCK, IRF1, SERPING1, H2-D1, TLR13, TLR2, TRIM56, C1QB, C1QA, UNC93B1, H2-Q7, NLRC5, ZC3HAV1, INPP5D, C1RA, LY86, EIF2AK2, IRGM1, PSMB8, PSMB9, BST2, BCL6, AXL, MYO1G, C1QC
GO:0045087~innate immune response	7.54 × 10^2^^2^	IFITM3, C1QB, SPON2, C1QA, CD84, CSF1, UNC93B1, NLRC5, OAS1A, TREM2, ZC3HAV1, IFIT1, IFIT3, IFIH1, C4B, CLEC7A, JAK3, TRIM21, B2M, HERC6, OASL2, C1RA, FCER1G, RSAD2, SYK, DDX58, LY86, CYBB, IRGM1, EIF2AK2, CYBA, BST2, FCGR1, NAIP2, HCK, TYROBP, AXL, IRF1, SERPING1, TLR13, TLR2, C1QC, TRIM56
GO:0006954~inflammatory response	5.15 × 10^14^	PTGFR, CCL12, CALCA, CXCL9, NCF1, CSF1, CXCL1, AIF1, PTGS1, C4B, CRHBP, CLEC7A, STAB1, SPP1, CCL3, C3AR1, CCL2, SLC11A1, LY86, CYBB, CYBA, TNFRSF1B, TNFRSF1A, P2RX7, CXCL10, NAIP2, HCK, BCL6, TNIP2, AXL, FAS, TLR13, TLR2
GO:0051607~defense response to virus	5.61 × 10^14^	APOBEC1, IFITM3, SPON2, CXCL9, RSAD2, UNC93B1, DDX58, NLRC5, EIF2AK2, OAS1A, ZC3HAV1, DDX60, IFIT1, IFIT3, IFIH1, BST2, CXCL10, IRF1, ITGAX, PMAIP1, TRIM56, OASL2
GO:0009615~response to virus	2.52 × 10^1^^2^	IFITM3, RSAD2, DDX58, EIF2AK2, OAS1A, ZC3HAV1, DDX60, IFIT1, IFIT3, IFIH1, BST2, CXCL10, BCL3, TLR13, OASL2
GO:0035458~cellular response to interferon-beta	2.65 × 10^9^	GBP6, IFI204, IRF1, IFI203, IGTP, IRGM2, IRGM1, IFIT1, GBP2, IFIT3, GBP3
GO:0032496~response to lipopolysaccharide	4.44 × 10^9^	SPON2, PTGFR, CEBPB, CXCL9, GCH1, SLC11A1, EIF2AK2, CXCL1, TNFRSF1B, LITAF, TNFRSF1A, P2RX7, CXCL10, CASP8, PENK, CASP1, ACP5, FAS, LGALS9, TLR2
GO:0050830~defense response to Gram-positive bacterium	1.53 × 10^8^	GBP6, H2-T23, GBP7, NCF1, GBP9, LYZ2, P2RX7, HCK, ACP5, GBP2, B2M, MYO1F, TLR2, GBP3
GO:0042832~defense response to protozoan	5.00 × 10^8^	GBP6, GBP7, GBP9, SLC11A1, BCL3, IRGM2, IRF8, GBP2, GBP3
GO:0071222~cellular response to lipopolysaccharide	6.62 × 10^8^	CX3CR1, GBP6, SPON2, CEBPB, ARG1, SERPINE1, TNFRSF1B, LITAF, ICAM1, CXCL10, SBNO2, PLSCR2, FCGR4, TNIP2, AXL, CCL2, IRF8, GBP2, B2M
GO:0006955~immune response	1.68 × 10^7^	CCL12, CXCL9, H2-K1, LIF, CXCL1, TNFRSF1B, CTSS, VAV1, TNFRSF1A, CXCL10, BLNK, CCL3, CCL2, FAS, IRF8, LCP2, FCGR2B, B2M, H2-D1, TLR2, OASL2
GO:0044406~adhesion of symbiont to host	2.44 × 10^7^	GBP6, GBP7, GBP9, GBP2, ICAM1, GBP3
GO:0071346~cellular response to interferon-gamma	4.42 × 10^7^	GBP6, CCL12, GBP7, GBP9, H2-Q7, CCL3, CCL2, GBP2, AIF1, GBP4, GBP3
GO:0030593~neutrophil chemotaxis	5.09 × 10^7^	FCGR3, CCL12, EDN2, FCER1G, SYK, SPP1, CCL3, CCL2, CXCL1, NCKAP1L, VAV1
GO:0042742~defense response to bacterium	5.22 × 10^7^	SPON2, CEBPB, FCER1G, SYK, NCF1, ANXA3, LYZ2, SLC11A1, H2-M3, H2-K1, IRGM2, TNFRSF1A, FCGR1, NAIP2, BCL3, STAB1, IRF8
GO:0042590~antigen processing and presentation of exogenous peptide antigen via MHC class I	5.57 × 10^7^	FCGR1, FCGR3, FCER1G, H2-K1, IFI30
GO:0032760~positive regulation of tumor necrosis factor production	1.22 × 10^6^	SASH3, H2-T23, SPON2, FCER1G, CCL3, CCL2, CYBA, LGALS9, TNFRSF1A, TLR2
GO:0009617~response to bacterium	2.91 × 10^6^	FCGR1, P2RX7, NCF1, SLC11A1, CASP1, IRF8, FCGR2B, TLR2
GO:0002474~antigen processing and presentation of peptide antigen via MHC class I	3.56 × 10^6^	H2-T23, H2-BL, H2-Q6, H2-Q7, H2-K1, H2-M3, B2M, H2-D1
GO:0006935~chemotaxis	1.16 × 10^5^	CX3CR1, CXCL10, CXCL9, CCL12, RAC2, C3AR1, CCL3, CCL2, NCKAP1L, LGALS9, DOCK2, CYR61
GO:0002479~antigen processing and presentation of exogenous peptide antigen via MHC class I, TAP-dependent	1.52 × 10^5^	H2-T23, H2-Q7, H2-K1, B2M, PSMB8, H2-D1, PSMB9
GO:0019221~cytokine-mediated signaling pathway	1.69 × 10^5^	CX3CR1, CCL12, EDN2, STAT3, CSF2RB, TNFRSF1A, CSF2RB2, SOCS3, CCL2, PTPN6, IL6ST, JAK3, CD44
GO:0010628~positive regulation of gene expression	1.75 × 10^5^	PTGFR, CSF1, DDX58, SLC11A1, SERPINE1, STAT3, PLAUR, LIF, SOX11, IKZF1, POU4F1, FGF2, TNFRSF1A, P2RX7, CCL3, CTSH, VIM, LGALS9, ATF3, CD44, NKX3-1, TLR2
GO:0001916~positive regulation of T cell mediated cytotoxicity	1.93 × 10^5^	P2RX7, H2-T23, H2-K1, H2-M3, B2M, H2-D1
GO:0050766~positive regulation of phagocytosis	2.60 × 10^5^	FCGR1, FCGR3, FCER1G, PROS1, SLC11A1, CYBA, DOCK2, FCGR2B
GO:0009636~response to toxic substance	2.89 × 10^5^	CDKN1A, PENK, CCL3, EIF2AK2, FAS, NEFL, AHR, NUPR1, SLC7A11, TLR2
GO:0007155~cell adhesion	3.58 × 10^5^	CX3CR1, LGALS3BP, SPON2, CD84, VWF, CD93, TNFAIP6, TNFRSF12A, FBLIM1, MCAM, PCDH8, CYR61, ICAM1, MFAP4, GPNMB, CHL1, PDPN, STAB1, SPP1, ITGAX, CD9, CTNNAL1, CD33, CD44
GO:0006909~phagocytosis	4.43 × 10^5^	HCK, ANXA3, AXL, SLC11A1, PLD4, IRF8, VAV1, MYO1G
GO:0045576~mast cell activation	7.08 × 10^5^	FCGR3, FCER1G, LCP2, CD48, FCGR2B
GO:0048246~macrophage chemotaxis	9.77 × 10^5^	CX3CR1, CCL12, EDN2, CCL3, CCL2
GO:0034341~response to interferon-gamma	9.84 × 10^5^	IFITM3, BST2, GCH1, SLC11A1, IRGM2, TRIM21
GO:0030335~positive regulation of cell migration	1.04 × 10^4^	TNFAIP6, CSF1, SEMA3C, MCAM, AIF1, CYR61, CXCL10, GPNMB, PDPN, C3AR1, CCL3, CTSH, ROR2, MYO1F
GO:0050729~positive regulation of inflammatory response	1.54 × 10^4^	CCL12, SERPINE1, CCL3, CCL2, CTSS, TNFRSF1A, TLR2, TGM2
GO:0045730~respiratory burst	2.05 × 10^4^	NCF1, SLC11A1, CYBB, CYBA
GO:0043029~T cell homeostasis	2.36 × 10^4^	P2RX7, PMAIP1, FAS, NCKAP1L, AHR, JAK3
GO:0042771~intrinsic apoptotic signaling pathway in response to DNA damage by p53 class mediator	2.36 × 10^4^	CDKN1A, IFI204, BCL3, SHISA5, PMAIP1, NUPR1
GO:0045071~negative regulation of viral genome replication	2.75 × 10^4^	IFITM3, BST2, RSAD2, EIF2AK2, ZC3HAV1
GO:0008285~negative regulation of cell proliferation	2.92 × 10^4^	IFITM3, CDKN1A, IGFBP3, STAT3, LIF, EIF2AK2, DHCR24, FGF2, IFIT3, MYO16, RUNX1, BCL6, IRF1, INPP5D, CD9, ROR2, NKX3-1, SKAP2, TLR2
GO:0043615~astrocyte cell migration	3.24 × 10^4^	CCL12, HEXB, CCL3, CCL2
GO:0007568~aging	3.72 × 10^4^	CALCA, ARG1, STAT3, PENK, SERPING1, CCL2, APOD, TIMP1, TNFRSF1B, LITAF, FGF2, CTSC
GO:0071407~cellular response to organic cyclic compound	4.58 × 10^4^	P2RX7, MSR1, CEBPB, CCL12, CASP8, STAT3, CCL3, CYBA
GO:0071347~cellular response to interleukin-1	6.78 × 10^4^	CEBPB, CCL12, IRF1, SERPINE1, CCL3, CCL2, ICAM1, NKX3-1
GO:0030168~platelet activation	7.08 × 10^4^	ENTPD1, VWF, SYK, AXL, ADRA2C, VAV1
GO:0045944~positive regulation of transcription from RNA polymerase II promoter	8.08 × 10^4^	CSRNP1, CEBPB, CEBPD, HEXB, NLRC5, IKZF1, FGF2, CYR61, SBNO2, CCL3, JAK3, ZBTB7C, NKX3-1, WWTR1, EGR2, DDX58, SLC11A1, STAT3, ARID5A, LIF, SOX11, POU4F1, POU4F2, RUNX1, TNFRSF1A, HOXB9, TNIP2, IRF1, BCL3, CAPRIN2, FOSB, ATF3, TLR2, CREB5
GO:0043066~negative regulation of apoptotic process	8.49 × 10^4^	PTGFR, CDKN1A, STAT3, PLAUR, EIF2AK2, DHCR24, AIF1, POU4F1, CYR61, IFIT3, TNFRSF1A, BTC, HCK, SOCS3, BCL6, AXL, BCL3, SPP1, CTSH, FAS, NCKAP1L, TIMP1, CD44
GO:0008217~regulation of blood pressure	9.63 × 10^4^	CALCA, EDN2, GCH1, C3AR1, CYBA, AHR, PTGS1
GO:0071356~cellular response to tumor necrosis factor	9.78 × 10^4^	CRHBP, CCL12, CALCA, IRF1, CCL3, CCL2, CYBA, ICAM1, NKX3-1
GO:0035457~cellular response to interferon-alpha	1.20 × 10^3^	IFI204, AXL, IFIT1, IFIT3
GO:0043065~positive regulation of apoptotic process	1.45 × 10^3^	HRK, TNFRSF12A, GADD45B, IGFBP3, EIF2AK2, POU4F1, CYR61, P2RX7, CASP8, BCL6, INPP5D, CASP1, PMAIP1, FAS, NUPR1, TGM2
GO:0097191~extrinsic apoptotic signaling pathway	6.69 × 10^3^	P2RX7, CASP8, TNFRSF12A, FAS, TNFRSF1B
GO:0006915~apoptotic process	9.46 × 10^3^	HRK, CSRNP1, TNFRSF12A, NCF1, GADD45B, SHISA5, GDF6, LITAF, TNFRSF1A, RNF144B, NAIP2, CASP8, TNIP2, IRF1, INPP5D, CASP1, PMAIP1, FAS, MAP3K8, XAF1
GO:0042127~regulation of cell proliferation	9.50 × 10^3^	APOBEC1, CXCL10, HCK, CXCL9, BCL6, SERPINE1, TCF7, FAS, TNFRSF1B, TNFRSF1A, PTGS1
GO:0042981~regulation of apoptotic process	1.16 × 10^2^	HRK, CASP8, BCL3, CASP1, PMAIP1, EIF2AK2, FAS, TNFRSF1B, GDF6, TNFRSF1A
GO:0048678~response to axon injury	2.07 × 10^2^	ARG1, APOD, FGF2, AIF1
GO:0043524~negative regulation of neuron apoptotic process	2.89 × 10^2^	CEBPB, CCL12, CHL1, AXL, NEFL, CCL2, POU4F1, NES

GO: Gene Ontology. Only results with false discovery rate < 0.05 and apoptosis-related processes are listed.

**Table 3:** KEGG pathway enrichment results.

Term	*p*-Value	Genes
mmu04380:Osteoclast differentiation	3.44 × 10^9^	SYK, NCF1, CSF1, CYBB, CYBA, TREM2, TNFRSF1A, FCGR1, SOCS3, FCGR3, TYROBP, FCGR4, BLNK, ACP5, FOSB, LCP2, FCGR2B, IRF9
mmu04145:Phagosome	1.08 × 10^8^	H2-T23, MSR1, C1RA, H2-BL, NCF1, H2-Q6, H2-Q7, H2-M3, H2-K1, TAP1, CYBA, CTSS, FCGR1, FCGR3, CLEC7A, TUBB3, FCGR4, FCGR2B, H2-D1, TLR2
mmu04668:TNF signaling pathway	2.29 × 10^8^	CEBPB, CCL12, CSF1, LIF, CXCL1, TNFRSF1B, TNFRSF1A, ICAM1, CXCL10, SOCS3, CASP8, BCL3, CCL2, FAS, MAP3K8, CREB5
mmu05168:Herpes simplex infection	5.17 × 10^8^	H2-T23, CCL12, H2-BL, DDX58, H2-Q6, H2-Q7, H2-M3, H2-K1, EIF2AK2, OAS1A, TAP1, IFIT1, TNFRSF1A, IFIH1, SOCS3, CASP8, CCL2, FAS, IRF9, H2-D1, TLR2
mmu05169:Epstein-Barr virus infection	7.42 × 10^8^	H2-T23, ENTPD1, CDKN1A, H2-BL, SYK, DDX58, H2-Q6, H2-Q7, H2-M3, STAT3, H2-K1, EIF2AK2, ICAM1, VIM, JAK3, H2-D1, CD44
mmu04610:Complement and coagulation cascades	1.31 × 10^7^	C1QB, C1QA, C1RA, VWF, PROS1, SERPINE1, PLAUR, PLAT, C4B, C3AR1, SERPING1, A2M, C1QC
mmu05150:Staphylococcus aureus infection	1.50 × 10^7^	FCGR1, C1QB, C4B, C1QA, FCGR3, C1RA, FCGR4, C3AR1, FCGR2B, ICAM1, C1QC
mmu04612:Antigen processing and presentation	1.65 × 10^5^	H2-T23, H2-BL, H2-Q6, H2-Q7, H2-K1, H2-M3, TAP1, IFI30, B2M, CTSS, H2-D1
mmu05203:Viral carcinogenesis	2.12 × 10^5^	H2-T23, EGR2, CDKN1A, H2-BL, SYK, H2-Q6, H2-Q7, H2-M3, STAT3, H2-K1, EIF2AK2, CASP8, PMAIP1, IL6ST, JAK3, IRF9, H2-D1, CREB5
mmu05142:Chagas disease (American trypanosomiasis)	2.23 × 10^5^	C1QB, GNA14, C1QA, CCL12, CASP8, SERPINE1, CCL3, FAS, CCL2, TNFRSF1A, C1QC, TLR2
mmu05164:Influenza A	3.64 × 10^5^	CCL12, RSAD2, DDX58, MX2, EIF2AK2, OAS1A, TNFRSF1A, ICAM1, IFIH1, CXCL10, SOCS3, CASP1, CCL2, FAS, IRF9
mmu04060:Cytokine-cytokine receptor interaction	3.86 × 10^5^	CX3CR1, IL15RA, CCL12, CXCL9, TNFRSF12A, CSF1, LIF, CXCL1, CSF2RB, OSMR, TNFRSF1B, TNFRSF1A, CSF2RB2, CXCL10, CCL3, CCL2, FAS, IL6ST
mmu05416:Viral myocarditis	7.61 × 10^5^	H2-T23, CASP8, H2-BL, H2-Q6, H2-Q7, H2-K1, H2-M3, RAC2, H2-D1, ICAM1
mmu04666:Fc gamma R-mediated phagocytosis	1.23 × 10^4^	FCGR1, HCK, NCF1, SYK, MYO10, INPP5D, RAC2, DOCK2, FCGR2B, VAV1
mmu04650:Natural killer cell mediated cytotoxicity	1.41 × 10^4^	TYROBP, FCER1G, SYK, FCGR4, RAC2, FAS, PTPN6, LCP2, CD48, VAV1, ICAM1
mmu04062:Chemokine signaling pathway	1.60 × 10^4^	CX3CR1, CCL12, CXCL9, NCF1, STAT3, CXCL1, VAV1, CXCL10, HCK, CCL3, RAC2, GNB3, CCL2, DOCK2, JAK3
mmu05332:Graft-versus-host disease	1.69 × 10^4^	H2-T23, H2-BL, H2-Q6, H2-Q7, H2-K1, H2-M3, FAS, H2-D1
mmu05330:Allograft rejection	2.72 × 10^4^	H2-T23, H2-BL, H2-Q6, H2-Q7, H2-K1, H2-M3, FAS, H2-D1
mmu05133:Pertussis	2.77 × 10^4^	C1QB, C4B, C1QA, C1RA, IRF1, CASP1, SERPING1, IRF8, C1QC
mmu04940:Type I diabetes mellitus	5.14 × 10^4^	H2-T23, H2-BL, H2-Q6, H2-Q7, H2-K1, H2-M3, FAS, H2-D1
mmu05152:Tuberculosis	7.21 × 10^4^	CEBPB, FCER1G, SYK, CTSS, TNFRSF1A, FCGR1, FCGR3, CASP8, CLEC7A, FCGR4, ITGAX, FCGR2B, TLR2
mmu05144:Malaria	7.51 × 10^4^	CCL12, CCL2, HBB-B1, HBA-A2, HBA-A1, ICAM1, TLR2
mmu04115:p53 signaling pathway	8.25 × 10^4^	CDKN1A, CASP8, GADD45B, IGFBP3, SERPINE1, SHISA5, PMAIP1, FAS
mmu04662:B cell receptor signaling pathway	1.07 × 10^3^	CD72, SYK, INPP5D, BLNK, RAC2, PTPN6, FCGR2B, VAV1
mmu05162:Measles	1.13 × 10^3^	IFIH1, DDX58, MX2, STAT3, EIF2AK2, FAS, OAS1A, FCGR2B, JAK3, IRF9, TLR2
mmu05320:Autoimmune thyroid disease	1.17 × 10^3^	H2-T23, H2-BL, H2-Q6, H2-Q7, H2-K1, H2-M3, FAS, H2-D1
mmu04630:Jak-STAT signaling pathway	1.83 × 10^3^	CSF2RB2, IL15RA, SOCS3, STAT3, LIF, PTPN6, CSF2RB, IL6ST, OSMR, JAK3, IRF9
mmu05140:Leishmaniasis	3.39 × 10^3^	FCGR1, FCGR3, NCF1, FCGR4, CYBA, PTPN6, TLR2
mmu05160:Hepatitis C	4.04 × 10^3^	SOCS3, CDKN1A, DDX58, IRF1, STAT3, EIF2AK2, OAS1A, IFIT1, IRF9, TNFRSF1A
mmu04142:Lysosome	6.97 × 10^3^	HEXB, SLC11A1, LAPTM5, ACP5, CTSH, CD68, LITAF, CTSS, CTSC
mmu05143:African trypanosomiasis	8.14 × 10^3^	FAS, HBB-B1, HBA-A2, HBA-A1, ICAM1
mmu04621:NOD-like receptor signaling pathway	9.72 × 10^3^	NAIP2, CCL12, CASP8, CASP1, CCL2, CXCL1
mmu05166:HTLV-I infection	1.10 × 10^2^	H2-T23, IL15RA, EGR2, CDKN1A, H2-BL, H2-Q6, H2-Q7, H2-M3, H2-K1, TNFRSF1A, ICAM1, JAK3, ATF3, H2-D1
mmu05323:Rheumatoid arthritis	1.13 × 10^2^	CCL12, CSF1, CCL3, CCL2, ACP5, ICAM1, TLR2
mmu05161:Hepatitis B	1.92 × 10^2^	IFIH1, EGR2, CDKN1A, CASP8, DDX58, STAT3, FAS, CREB5, TLR2
mmu04664:Fc epsilon RI signaling pathway	1.98 × 10^2^	FCER1G, SYK, INPP5D, RAC2, LCP2, VAV1
mmu04620:Toll-like receptor signaling pathway	2.87 × 10^2^	CXCL10, CXCL9, CASP8, SPP1, CCL3, MAP3K8, TLR2
mmu04611:Platelet activation	3.12 × 10^2^	FCER1G, VWF, SYK, COL5A2, TBXAS1, PRKG2, LCP2, PTGS1
mmu05134:Legionellosis	4.17 × 10^2^	NAIP2, CASP8, CASP1, CXCL1, TLR2
mmu04210:Apoptosis	4.88 × 10^2^	CSF2RB2, CASP8, FAS, CSF2RB, TNFRSF1A

KEGG: Kyoto Encyclopedia of Genes and Genomes.

**Table 4:** Hub genes identified by topological characteristics.

Genes	DC	Genes	EC	Genes	LAC	Genes	BC	Genes	CC
*Cxcl10*	77	*Tlr2*	0.16	*Ifit3*	28.38	*Stat3*	6332.91	*Cxcl10*	0.044
*Tlr2*	73	*Tyrobp*	0.16	*Ifit1*	28.13	*Cd44*	5426.13	*Stat3*	0.044
*Tyrobp*	70	*Cxcl10*	0.16	*Irf9*	27.79	*Cxcl10*	4681.39	*Tlr2*	0.043
*Itgax*	66	*Itgax*	0.16	*Rtp4*	27.67	*Tlr2*	4583.62	*Ccl2*	0.043
*Stat3*	64	*Aif1*	0.15	*Oasl2*	27.58	*Aif1*	4057.10	*Cd44*	0.043
*Aif1*	62	*Cd68*	0.15	*Irgm1*	27.47	*Vwf*	3852.56	*Aif1*	0.043
*Cd68*	60	*Irf8*	0.14	*Irgm2*	27.46	*Atf3*	3484.15	*Itgax*	0.043
*Ccl2*	60	*Fcgr2b*	0.14	*Gbp2*	27.11	*Vim*	3158.54	*Cd68*	0.043
*Ctss*	58	*Ctss*	0.14	*Parp14*	26.92	*Ccl2*	3102.06	*Cxcl9*	0.043
*Cd44*	57	*Cxcl9*	0.13	*Gbp3*	26.69	*Fgf2*	2981.26	*Tyrobp*	0.043
*Fcgr2b*	55	*Fcgr1*	0.13	*Rnf213*	26.37	*Entpd1*	2352.07	*Icam1*	0.043
*Cxcl9*	55	*Nckap1l*	0.13	*Fcer1g*	26.27	*Igfbp3*	2348.79	*Irf8*	0.043
*C1qa*	55	*C1qa*	0.13	*Rsad2*	26.15	*Antxr2*	2238	*C1qa*	0.043
*Irf8*	54	*Ccl2*	0.13	*Tyrobp*	26	*Tnnt2*	2238	*Cxcl1*	0.043
*Nckap1l*	54	*Casp1*	0.12	*Parp9*	25.94	*C1qa*	2156.60	*Csf1*	0.043
*Irf1*	54	*Cybb*	0.12	*Herc6*	25.89	*Itgax*	2130.30	*Fcgr2b*	0.043
*Ifih1*	54	*Fcer1g*	0.12	*Ddx60*	25.83	*Socs3*	2039.41	*Fgf2*	0.043
*Ddx58*	54	*Cd53*	0.12	*Nckap1l*	25.48	*Timp1*	1996.1	*Casp1*	0.043
*Mx1*	50	*Fcgr4*	0.12	*Pld4*	25.44	*Cxcl1*	1920.96	*Timp1*	0.043
*C1qb*	48	*Lyz2*	0.12	*Cd53*	25.32	*Cxcl9*	1830.66	*Cybb*	0.043
*Casp1*	47	*Stat3*	0.12	*Trim30a*	25.30	*C1qb*	1772.68	*Lyz2*	0.043
*Syk*	47	*Irf1*	0.11	*Gbp6*	25.24	*Tyrobp*	1763.02	*Ctss*	0.043
*Icam1*	47	*Ifih1*	0.11	*Ifih1*	25.15	*Spp1*	1704.59	*Fcgr1*	0.043
*Ptpn6*	47	*Ly86*	0.11	*Mx1*	25.08	*Bst1*	1680	*Irf1*	0.043
*Fcgr1*	46	*H2-K1*	0.11	*Cxcl10*	24.80	*Irf1*	1619.14	*H2-K1*	0.043
*Lyz2*	46	*Ddx58*	0.11	*Igtp*	24.77	*Irf8*	1564.14	*Ccl3*	0.043
*Ly86*	46	*Pld4*	0.11	*Irf1*	24.59	*Cd68*	1519.08	*Serpine1*	0.043
*H2-K1*	46	*Mx1*	0.11	*Ctss*	24.24	*C4b*	1491.62	*Ddx58*	0.043
*Cybb*	45	*Fcgr3*	0.11	*Gbp7*	24.23	*Arg1*	1475.19	*Psmb8*	0.043
*Fcgr4*	45	*Syk*	0.11	*Fcgr1*	24.23	*Mx1*	1474.78	*Syk*	0.043
*Ifit1*	45	*Cd48*	0.11	*Cd68*	23.6	*Cyr61*	1448.63	*Socs3*	0.043
*Fcer1g*	44	*C1qb*	0.11	*Fcgr2b*	23.56	*Ddx58*	1429.51	*Spp1*	0.043
*Cd53*	44	*Vav1*	0.101	*Ly86*	23.52	*Lyz2*	1427.96	*Mx1*	0.043
*Psmb8*	44	*Cd44*	0.101	*Samd9l*	23.36	*Gnb3*	1422.19	*B2m*	0.043
*Pld4*	43	*Slc11a1*	0.101	*Itgax*	23.33	*Csf1*	1404.39	*Gfap*	0.043
*Fcgr3*	43	*Icam1*	0.101	*Ddx58*	23.22	*Myo1g*	1373.28	*Arg1*	0.043
*Lcp2*	43	*Hck*	0.101	*Aif1*	22.90	*Ctsc*	1337.92	*Fcgr4*	0.043
*Trim30a*	43	*Ptpn6*	0.10	*Trim21*	22.85	*Lcp2*	1322.43	*Vwf*	0.043
*Cxcl1*	43	*Ifit1*	0.10	*Hck*	22.70	*Icam1*	1297.18	*Vav1*	0.043
*Oasl2*	43	*Psmb8*	0.10	*Fcgr3*	22.60	*Serpine1*	1273.40	*C1qb*	0.043
*Cd48*	42	*Lcp2*	0.10	*Tlr2*	22.52	*Gbp7*	1255.33	*Ptpn6*	0.043
*Vav1*	42	*Trim30a*	0.10	*Laptm5*	22.4	*Ctss*	1219.90	*Cd48*	0.043
*Ifit3*	42	*Rac2*	0.09	*Fcgr4*	22	*Cdkn1a*	1208.70	*C3ar1*	0.043
*Rtp4*	42	*Csf1*	0.09	*Rac2*	21.68	*B2m*	1202.51	*Nckap1l*	0.043
*Csf1*	41	*Ccl3*	0.09	*Cd48*	21.67	*Hbb-bs*	1200.55	*Cx3cr1*	0.043
*Rsad2*	41	*C3ar1*	0.09	*Cybb*	21.56	*Pou4f1*	1195.79	*Ifih1*	0.043
*Psmb9*	40	*Cxcl1*	0.09	*Bst2*	21.44	*Hexb*	1156.12	*Psmb9*	0.043
*C1qc*	40	*Laptm5*	0.09	*Cxcl9*	21.13	*Pola2*	1154.03	*Cd53*	0.043
*C3ar1*	39	*Psmb9*	0.09	*Eif2ak2*	20.93	*Lrrc2*	1126	*Lcp2*	0.043
*Igtp*	39	*C1qc*	0.09	*Irf8*	20.85	*Prkg2*	1126	*Ly86*	0.043
*Irf9*	39	*Trem2*	0.09	*Lyz2*	20.70	*Ahr*	1124	*Fcgr3*	0.043
*Parp14*	39	*Irgm1*	0.09	*Psmb8*	20.5	*Map3k8*	1124	*Clec7a*	0.043
*Irgm1*	38	*Igtp*	0.09	*Slc11a1*	20.27	*Irx4*	1124	*Fas*	0.043
*Gbp2*	38	*Oasl2*	0.09	*C1qa*	20.22	*Nes*	1109.62	*C1qc*	0.043
*Rnf213*	38	*Gbp2*	0.08	*Psmb9*	20.15	*Fcgr2b*	1042.78	*Atf3*	0.043
*B2m*	38	*Ifit3*	0.08	*Cd52*	20.08	*Nckap1l*	1029.29	*Trem2*	0.043
*Slc11a1*	37	*Rtp4*	0.08	*Myo1f*	19.48	*Casp1*	1026.18	*Slc11a1*	0.043
*Hck*	37	*Irf9*	0.08	*Vav1*	19.43	*C3ar1*	979.36	*Cyba*	0.043
*Rac2*	37	*Rsad2*	0.08	*Casp1*	19.23	*Psmb8*	891.35	*Lcp1*	0.043
*Irgm2*	37	*Cyba*	0.08	*Ptpn6*	19.23	*Ifih1*	883.81	*Hck*	0.043

DC: degree centrality; EC: eigenvector centrality; LAC: local average connectivity; BC: betweenness centrality; CC: closeness centrality.

**Table 5:** Modules screened from the PPI network.

Cluster	Score	Nodes	Edges	Node IDs
1	27.357	29	383	*Herc6*, *Irf1*, *Irf9*, *Ifih1*, *Oasl2*, *Gbp7*, *Parp9*, *Gbp2*, *Ddx60*, *Rsad2, Psmb9*, *Bst2*, *Gbp3*, *Irgm2*, *Rnf213*, *Ifit1*, *Trim21, Samd9l, Gbp6*, *Trim30a*, *Irgm1*, *Eif2ak2*, *Ddx58*, *Cxcl10*, *Rtp4*, *Igtp*, *Parp14*, *Psmb8*, *Ifit3*
2	20.968	32	325	*Ptpn6*, *Ly86*, *Ifi203*, *Lyz2*, *Ctss*, *Cybb*, *Inpp5d*, *Tyrobp*, *Irf8*, *Laptm5*, *Ifi204*, *Csf2rb2*, *C1qa*, *Cxcl9*, *Cd68*, *Fcgr3*, *Nckap1l*, *Hck*, *Lcp2*, *Slc11a1*, *Gbp4*, *Fcer1g*, *Cd52*, *Cd48*, *Fcgr1*, *Rac2*, *Sash3*, *Pld4*, *Aif1*, *Myo1g*, *Cd53*, *Fcgr4*
3	9.692	27	126	*Stat3*, *H2-Q6*, *Tlr2*, *Cd93*, *Casp1*, *Icam1*, *Myo1f*, *Arg1*, *Timp1*, *Ifitm3*, *Fcgr2b*, *Cyba*, *Ccl2*, *Unc93b1*, *Itgax*, *Csf1*, *C1qc*, *H2-Q7, Ccl3*, *C1qb*, *Tnfrsf1a*, *C3ar1*, *Vav1*, *H2-D1*, *Arhgap30*, *H2-T23*, *H2-M3*
4	4.8	6	12	*Gfap*, *Pvalb*, *Calb2*, *Pou4f1*, *Tubb3*, *Lif*
5	4.4	16	33	*Cx3cr1*, *Fas*, *H2-K1*, *Syk*, *Fgf2*, *Serpine1*, *Cd44*, *Ccl12*, *Ikzf1*, *Lcp1*, *Wdfy4*, *B2m*, *Cxcl1*, *Cyth4*, *Spp1*, *Trem2*

PPI: protein–protein network.

**Table 6:** Potential drugs that reversed the expression of genes induced by nanoparticles.

CMap Name	Mean	Enrichment	*p*-Value
5182598	−0.66	−0.97	2.03 × 10^3^
Tocainide	−0.58	−0.92	6.00 × 10^5^
NU-1025	−0.38	−0.88	2.88 × 10^2^
Harpagoside	−0.37	−0.88	5.20 × 10^4^
Cloxacillin	−0.24	−0.83	1.55 × 10^3^
Prestwick-1103	−0.26	−0.80	3.18 × 10^3^
Benzathine benzylpenicillin	−0.27	−0.79	4.08 × 10^3^
Folic acid	−0.25	−0.78	4.44 × 10^3^
Prestwick-967	−0.50	−0.77	5.61 × 10^3^
Timolol	−0.25	−0.77	5.89 × 10^3^
Prestwick-675	−0.26	−0.77	6.15 × 10^3^
Indoprofen	−0.21	−0.75	7.70 × 10^3^
Atractyloside	−0.33	−0.71	4.45 × 10^3^
Arcaine	−0.41	−0.69	2.01 × 10^2^
Retrorsine	−0.38	−0.69	2.14 × 10^2^
Prestwick-689	−0.38	−0.68	2.32 × 10^2^
Fursultiamine	−0.51	−0.67	2.72 × 10^2^
Isometheptene	−0.23	−0.65	3.44 × 10^2^
Diphenylpyraline	−0.29	−0.56	2.94 × 10^2^
Vincamine	−0.20	−0.54	3.79 × 10^2^

CMap: Connectivity Map.

## Data Availability

All data were downloaded from the GEO database (GSE49371, GSE49048, GSE55228, GSE120257, GSE131486, GSE173233, GSE151610, GSE135844, GSE122270; http://www.ncbi.nlm.nih.gov/geo/).

## Abbreviations

NPs	nanoparticles
Ag	silver
Au	gold
Si	silicate
IL	interleukin
TNF	tumor necrosis factor
TiO2	titanium dioxide
NCBI	National Center for Biotechnology Information
GEO	Gene Expression Omnibus
PBS	phosphate buffer saline
PTC	presumptive therapeutic concentration
ONC	optic nerve crush
FC	fold change
DE-lncRNAs	differentially expressed long non-coding RNAs
DE-mRNAs	differentially expressed messenger RNAs
GO	Gene Ontology
KEGG	Kyoto Encyclopedia of Genes and Genomes
DAVID	Database for Annotation, Visualization and Integrated Discovery
PPI	Protein–protein interaction
DC	degree centrality
EC	eigenvector centrality
LAC	local average connectivity
BC	betweenness centrality
CC	closeness centrality
MCODE	Molecular Complex Detection
ceRNAs	competing endogenous RNAs
miRNAs	microRNAs
CMap	Connectivity Map
CTD	Comparative Toxicogenomics Database
SOCS3	suppressor of cytokine signaling 3
STAT3	signal transducer and activator of transcription 3
FAS	Fas cell surface death receptor
CASP1	caspase 1
CCL2	C-C motif chemokine ligand 2
CXCL10	C-X-C motif chemokine ligand 10
IRF8	interferon regulatory factor 8
SYK	spleen tyrosine kinase
TLR	toll-like receptor
GDF6	growth differentiation factor 6
CRHBP	corticotropin-releasing hormone-binding protein
NF-κB	nuclear factor κB
